# Symptom Duration and Surgeon Volume: Impact on Early Laparoscopic Cholecystectomy for Acute Cholecystitis

**DOI:** 10.7759/cureus.47517

**Published:** 2023-10-23

**Authors:** Yasin Güneş, İksan Taşdelen, Anıl Ergin, Ahmet Çakmak, Ali Cihan Bilgili, Anıl Bayram, Mehmet T Aydın

**Affiliations:** 1 General Surgery Department, Fatih Sultan Mehmet Training and Research Hospital, Istanbul, TUR; 2 General Surgery, Sinop Ayancık State Hospital, Sinop, TUR

**Keywords:** low-volume surgeons, high-volume surgeons, early laparoscopic cholecystectomy, symptom duration, acute cholecystitis

## Abstract

Background: The ‘golden 72 hours’ rule from the onset of symptoms still applies in laparoscopic cholecystectomy for acute cholecystitis. This rule has been discussed with increasing experience in laparoscopic surgery in recent years.

Objective: This study aims to determine the optimal symptom duration based on the surgeon's volume when deciding on early laparoscopic cholecystectomy for acute cholecystitis.

Materials and Methods: The patients were categorized into two groups: Group 1 (≤3 days) and Group 2 (>3 days) based on the symptom duration, and high-volume surgeons (performing >100 laparoscopic cholecystectomies in a year) and low-volume surgeons (performing <100 laparoscopic cholecystectomies in a year) based on the surgeon volume. All surgeons had received advanced training in laparoscopic surgery.

Results: There was no statistical difference in postoperative outcomes between groups, except for a few data (p>0.05). The operative time was longer in Group 2, the postoperative hospital stay was longer for low-volume surgeons than for high-volume surgeons after three days, and operative time was longer after three days than the first three days in low-volume surgeons (p<0.05).

Conclusions: Early laparoscopic cholecystectomy may be recommended for acute cholecystitis with symptom duration of more than three days, regardless of the surgeon volume, as long as they are competent in laparoscopic surgeries.

## Introduction

Gallstone disease affects approximately 10-15% of the adult population [1]. Each year, around 1-4% of asymptomatic patients develop symptoms, particularly acute cholecystitis (AC) [2]. Initially, laparoscopic cholecystectomy (LC) was contraindicated for AC during the early days of laparoscopic surgery. Early studies reported high mortality rates and bile duct injuries associated with LC [3]. However, as experience in laparoscopic surgery increased over the years, early laparoscopic cholecystectomy (ELC) has demonstrated superiority over delayed laparoscopic cholecystectomy, as it reduced hospital stays and had similar postoperative outcomes [4]. Currently, LC is considered the gold standard for treating AC [5].

The optimal timing of AC remains a topic of debate [6]. Traditionally, it has been believed that performing LC more than 72 hours after the onset of symptoms increases the risk of bile duct injury and conversion to open cholecystectomy [7]. The Tokyo 2013 guidelines confirmed this recommendation [8]. However, delaying surgery in patients with symptom duration exceeding 72 hours results in 17.5% of cases requiring emergency surgery due to conservative treatment failure or symptom recurrence after discharge, leading to prolonged hospital stays [9]. The symptom duration has started to be discussed with the increasing experience in laparoscopic surgery in recent years. Several studies now suggest that the symptom duration should be extended to seven days or the symptom duration may not be a significant factor in ELC. Finally, these viewpoints have even been incorporated into the guidelines [10,11].

However, limited research is available that evaluates surgeon experience or the relationship between surgeon experience and symptom duration in ELC [12-15]. Surgeon experience plays a crucial role in determining outcomes in laparoscopic surgery. Kortram et al. [13] found that surgeons who performed a minimum of 50 laparoscopic operations per year had lower conversion rates and shorter operating times in AC cases. Dankevort et al. [14] reported that gastrointestinal surgeons who conducted over 20 complex laparoscopic gastrointestinal surgeries annually had lower conversion and similar complication rates. Similarly, Abelson et al. [15] found that complication rates among surgeons trained in advanced laparoscopic surgery were similar to those of other surgeons, and their conversion rates were low. These three studies aimed to compare surgeons who received training in laparoscopic surgery with those who did not receive such training. In the study conducted by Di Martino et al. [12], all surgeons included in the study had received training in laparoscopic surgery. The findings revealed that complication rates were similar between patients with symptom duration within the first seven days and those with symptom duration beyond seven days. Additionally, low-volume centers were observed to increase complication and conversion rates if the symptom duration was beyond seven days.

Our study aimed to evaluate the influence of three key factors in ELC for AC: the duration from symptom onset to surgery, the surgeon volume, and the potential relationship between these factors.

## Materials and methods

This study was approved by the Ethics Committee of the University of Health Sciences, Istanbul Fatih Sultan Mehmet Training and Research Hospital, and was conducted in accordance with the principles of the Declaration of Helsinki (approval code: FSMEAH-KAEK 2022/147).

Study design

We retrospectively reviewed all patients hospitalized for gallstone-related diseases at the general surgery clinic between July 2011 and July 2021. The study included all adult patients aged 18 years and older hospitalized with a diagnosis of acute calculous cholecystitis and subsequently underwent LC. The diagnosis of AC was based on the Tokyo 2018 guidelines, which considered the presence of local inflammation findings (such as right upper quadrant pain, tenderness, palpable mass, and Murphy's sign), systemic inflammation findings (such as fever, elevated white blood cell (WBC) count, and elevated C-reactive protein (CRP) levels), and radiological findings (including gallbladder wall thickness ≥4 mm, pericholecystic fluid on imaging studies like upper abdomen ultrasonography, magnetic resonance cholangiopancreatography, or computed tomography, or sonographic Murphy positivity) [10]. Patients who had experienced multiple AC attacks, gallstone-related jaundice, or acute pancreatitis in conjunction with AC, as well as those who had a history of gallstone-related jaundice or acute pancreatitis prior to the AC episode, were excluded from the study; this exclusion criterion aimed to minimize potential variations in intraoperative surgical difficulty. Therefore, the study specifically focused on patients who presented with their first episode of AC and subsequently underwent LC.

Groups

We calculated the duration from the onset of symptoms to hospital admission, the interval between admission and surgery, as well as the combined total of these two time periods for each patient. We divided the patients into two groups based on the time from symptom onset to surgery. Patients who underwent surgery within the first three days were categorized as Group 1, while those who had surgery after three days were categorized as Group 2. Furthermore, the surgeons at our clinic are specialized in three branches of general surgery: breast-endocrine-hernia surgery, gastrointestinal surgery, and hepatopancreaticobiliary surgery. Elective gallbladder surgeries were performed by hepatopancreaticobiliary surgeons, each conducting over 100 laparoscopic cholecystectomies annually. However, AC surgery is considered an emergency procedure performed by the on-duty general surgeon from any branch. Consequently, we divided the surgeons into two groups. The high-volume surgeons (HVS) were the hepatopancreaticobiliary surgeons who performed elective gallbladder surgeries, while the low-volume surgeons (LVS) consisted of surgeons from other branches who did not perform elective gallbladder surgeries.

Preoperative characteristics and intraoperative findings

We collected a comprehensive set of data including the patient's age, gender, clinical, laboratory, radiological, and intraoperative findings. Clinical findings include the Charlson comorbidity index, anesthesia risk, symptom duration, and AC severity grade. Laboratory findings include WBC count, CRP, aspartate aminotransferase (AST), alanine aminotransferase (ALT), alkaline phosphatase (ALP), gamma-glutamyl transferase (GGT), total bilirubin (TBIL), and direct bilirubin (DBIL). Radiological findings include gallbladder wall thickness, gallbladder diameter, and the presence of pericholecystic fluid. Intraoperative findings include gallbladder wall edema, hydropic gallbladder, adhesions, gangrenous gallbladder, perforated gallbladder, partial cholecystectomy, and Parkland score. We calculated each patient's Charlson comorbidity index by scoring between 1 and 6 for age and comorbid diseases. The American Society of Anesthesiology risk classification was utilized to evaluate the anesthesia risk for each patient. Moreover, in accordance with the Tokyo 2018 guidelines, we classified AC into three grades: grade 1 (mild), grade 2 (moderate), and grade 3 (severe) [10]. The Parkland score was calculated by scoring the intraoperative findings of the gallbladder [16].

Postoperative outcomes

We documented various aspects related to the surgical procedures, including the types of surgery performed, complications encountered, duration of the operation, the need for intensive care treatment, readmission rates, length of hospital stays, and pathological findings. The surgical procedures conducted consisted of LC as well as cases where conversion to open cholecystectomy was required. Hospital stay was categorized into postoperative hospital stay and total hospital stay, taking into account the duration from admission to discharge. The recorded complications encompassed a range of issues including bile duct injury, visceral injury, bleeding, bile leakage, perihepatic collection, obstructive jaundice, acute pancreatitis, and gastrointestinal hemorrhage.

Statistical analysis

For statistical analysis, we utilized SPSS Software, version 25.0 (IBM Corp., Armonk, NY). Descriptive statistical methods were employed to evaluate the study data, including mean, standard deviation, frequency, and percentage. The Shapiro-Wilks test was used to assess the normality of quantitative parameters. Student t-test was utilized for comparing normally distributed parameters between groups, while the Mann-Whitney U test was employed for comparing non-normally distributed parameters. The comparison of qualitative data was performed using Pearson's Chi-square test and Fisher's exact test. Statistical significance was considered for p-values less than 0.05.

## Results

The patients diagnosed with acute calculous cholecystitis were 117. Group 1 comprised 49 patients, while Group 2 had 68 patients. The female gender accounted for 50.4% of the cases, with a mean age of 47.08 and a mean Charlson comorbidity index of 0.9 (p=0.391, p=0.755, p=0.061, respectively). There was a significant difference in the American Society of Anesthesiologists (ASA) scores between the two groups, with a higher proportion of ASA 3-4 patients in Group 2 (p=0.024). Additionally, Group 2 exhibited a higher incidence of Grade 2-3 AC (p<0.001). The average duration of symptoms from onset to diagnosis was 2.2 days, from diagnosis to surgery was 1.3 days, and from onset to surgery was 3.6 days (Table 1).

Regarding the laboratory findings, the mean WBC was 13*10^9^/uL (p=0.925), and the mean CRP level was 7.08 mg/L (p<0.001). The average AST level was 36.6 U/L (p=0.020), ALT level was 42 U/L (p=0.074), GGT level was 72.2 U/L (p=0.013), total bilirubin level was 0.7 mg/dL (p=0.140), and direct bilirubin level was 0.3 mg/dL (p=0.122). The mean amylase level was 53.1 U/L (p=0.588), and the mean lipase level was 23.1 U/L (p=0.697). On ultrasonography, the average gallbladder wall thickness was 4.4 mm (p=0.993), the mean gallbladder diameter was 3.3 cm (p=0.004), and the pericholecystic fluid rate was 29.9% (p=0.006). In terms of intraoperative findings, gallbladder wall edema was observed in 59.8% of the patients (p=0.305), hydropic gallbladder in 65% (p=0.745), adhesions in 49.6% (p=0.629), gangrenous gallbladder in 16.2% (p=0.133), perforated gallbladder in 9.4% (p=0.355), and subtotal cholecystectomy in 6% (p=0.040). While the majority of surgeries were performed by HVS in the first three days of symptom duration, the number of surgeries was equal among surgeons after three days (p=0.083) (Table 1).

**Table 1:** Preoperative characteristics and intraoperative findings ¹ Pearson chi-square test, ^2 ^Student-t test, ^3 ^Mann-Whitney U test, ^4 ^Fisher’s exact test, * p<0.05 significance, CCI: Charlson comorbidity index, ASA: American Society of Anesthesiologists, WBC: White blood cell, CRP: C-reactive protein, AST: Aspartate aminotransferase, ALT: Alanine aminotransferase, ALP: Alkaline phosphatase, GGT: Gamma-glutamyl transferase, TBIL: Total bilirubin, DBIL: Direct bilirubin, GB: Gallbladder

		Group 1 (n:49)	Group 2 (n:68)	Total (n:117)	p-value
Gender	Male	22(44.9)	36(52.9)	58(49.6)	^1^0.391
	Female	27(55.1)	32(47.1)	59(50.4)	
Age, mean (year)		46.6	47.4	47.08	^2^0.755
CCI, mean		0.5	1.2	0.9	^3^0.061
ASA score, n(%)	1-2	46(93.9)	52(78.8)	98(85.2)	^1^0.024*
	3-4	3(6.1)	14(21.2)	17(14.8)	
Grade, n(%)	1, mild	43(87.8)	36(52.9)	79(67.5)	^4^<0.001*
	2, moderate	5(10.2)	28(41.2)	33(28.2)	
	3, severe	1(2)	4(5.9)	5(4.3)	
Symptom duration, mean (days)	At diagnosis	0.8	3.2	2.2	^3^<0.001*
	After diagnosis	0.5	1.9	1.3	^3^<0.001*
	Total	1.3	5.2	3.6	^3^<0.001*
Laboratory findings	WBC, mean (10^9/uL)	12.9	13	13	^3^0.925
	CRP, mean (mg/L)	3.8	9.4	7.08	^3^<0.001^*^
	AST, mean (U/L)	39.5	34.5	36.6	^3^0.020^*^
	ALT, mean (U/L)	48	38	42	^3^0.074
	GGT, mean (U/L)	59.5	81.4	72.2	^3^0.013^*^
	TBIL, mean (mg/dL)	0.7	0.8	0.7	^3^0.140
	DBIL, mean (mg/dL)	0.2	0.3	0.3	^3^0.122
	Amylase, mean (U/L)	54.5	52	53.1	^3^0.588
	Lipase, mean (U/L)	23.7	22.7	23.1	^3^0.697
Radiological findings	Gallbladder wall thickness, mean (mm)	4.4	4.4	4.4	^3^0.993
	Gallbladder diameter, mean (cm)	3	3.6	3.3	^3^0.004^*^
	Pericholecystic fluid, n(%)	8(16.3)	27(39.7)	35(29.9)	^1^0.006*
Intraoperative findings	Edema in GB wall, n(%)	32(65.3)	38(55.9)	70(59.8)	^1^0.305
	Hydropic GB, n(%)	31(63.3)	45(66.2)	76(65)	^1^0.745
	Adhesions, n(%)	23(46.9)	35(51.5)	58(49.6)	^1^0.629
	Gangrenous GB, n(%)	5(10.2)	14(20.6)	19(16.2)	^1^0.133
	Perforated GB, n(%)	3(6.1)	8(11.8)	11(9.4)	^4^0.355
	Subtotal cholecystectomy, n(%)	0	7(10.3)	7(6)	^4^0.040^*^
	Parkland score, mean	2.8	3.2	3	^3^0.043*
Surgeons	Low-volume surgeons	18(36.7)	36(52.9)	54(46.2)	0.083^1^
	High-volume surgeons	31(63.3)	32(47.1)	63(53.8)	

The conversion rate to open cholecystectomy was 3.6%, and the overall complication rate was 1.7% in all patients. There was no significant statistical difference observed between the groups (p=1.000, p=0.509, respectively). The mean operative time was 69.7 minutes in Group 1 and 85 minutes in Group 2, showing a significant difference (p<0.001). The postoperative intensive care treatment rate was 2.6% (p=0.263), and the readmission rate was 4.3% among all patients (p=1.000). Neither of these rates exhibited statistical significance between the groups. The mortality rates were similar across both groups (p=1.000). The postoperative hospital stay did not differ significantly between the groups, with a mean duration of 2.4 days (p=0.199). The incidence of AC in pathology specimens was 20.4% in Group 1 and 39.7% in Group 2, indicating a significant difference (p=0.027). Overall, apart from the differences in operative times and pathology findings, there were no significant distinctions observed among the groups (Table 2).

**Table 2:** Intraoperative and postoperative outcomes ¹ Fisher’s exact test, ² Mann-Whitney U test, ^3^ Pearson chi-square test, * p<0.05 significance

	Group 1 (n:49)	Group 2 (n:68)	Total (n:117)	p-value
Conversion to open cholecystectomy, n(%)	2(4.1)	2(2.9)	4(3.4)	^1^1.000
Complications, n(%)	0	2(2.9)	2(1.7)	^1^0.509
Operative time, mean (minute)	69.7	85	78.6	^2^0.001*
Intensive care treatment, n(%)	0	3(4.4)	3(2.6)	^1^0.263
Readmission, n(%)	2(4.1)	3(4.4)	5(4.3)	^1^1.000
Mortality, n(%)	0	1(1.5)	1(0.9)	^1^1.000
Postoperative hospital stay, mean (days)	1.9	2.8	2.4	^2^0.199
Pathology (Acute cholecystitis), n(%)	10(20.4)	27(39.7)	37(31.6)	^3^0.027^*^

Table 3 and Table 4 compare the preoperative characteristics, intraoperative findings, and postoperative outcomes between the HVS and LVS. The analysis showed no statistically significant differences between the two groups in preoperative characteristics and most intraoperative findings. However, there were significant differences in the rates of subtotal cholecystectomy, with LVS having a rate of 11.1% compared to 1.6% in HVS (p=0.030). The Parkland score was 3.3 in LVS and 2.8 in HVS (p=0.008). Additionally, the incidence of gangrenous and perforated gallbladders was approximately two times higher in LVS, although these differences did not reach statistical significance (p=0.262, p=0.222, respectively).

**Table 3:** Preoperative characteristics and intraoperative findings among the surgeons ^1 ^Pearson Chi-Square test, ^2 ^Student t-test, ^3 ^Mann Whitney U test, ^4 ^Fisher’s exact test, * p<0.05 significance, CCI: Charlson comorbidity index, ASA: American Society of Anesthesiologists, WBC: White blood cell, CRP: C-reactive protein, AST: Aspartate aminotransferase, ALT: Alanine aminotransferase, ALP: Alkaline phosphatase, GGT: Gamma-glutamyl transferase, TBIL: Total bilirubin, DBIL: Direct bilirubin, GB: Gallbladder

		Low-volume surgeons (n:54)	High-volume surgeons (n:63)	p-value
Gender	Male	28(51.9)	30(47.6)	^1^0.648
	Female	26(48.1)	33(52.4)	
Age, mean (year)		48	46.3	^2^0.510
CCI, mean		1.05	0.8	^3^0.778
ASA score, n(%)	1-2	43(81.1)	55(88.7)	^1^0.254
	3-4	10(18.9)	7(11.3)	
Grade, n(%)	1, mild	34(63)	45(71.4)	^4^0.257
	2, moderate	16(29.6)	17(27)	
	3, severe	4(7.4)	1(1.6)	
Symptom duration, mean (days)	At diagnosis	2.4	2.1	^3^0.224
	After diagnosis	1.4	1.2	^3^0.656
	Total	3.9	3.3	^3^0.231
Laboratory findings	WBC, mean (10^9/uL)	12.7	13.1	^3^0.508
	CRP, mean (mg/L)	7.4	6.7	^3^0.692
	AST, mean (U/L)	29	42	^3^0.477
	ALT, mean (U/L)	36	49	^3^0.810
	GGT, mean (U/L)	81	64	^3^0.343
	TBIL, mean (mg/dL)	0.7	0.8	^3^0.366
	DBIL, mean (mg/dL)	0.3	0.3	^3^0.939
	Amylase, mean (U/L)	50	55	^3^0.203
	Lipase, mean (U/L)	22	23	^3^0.470
Radiological findings	Gallbladder wall thickness, mean (mm)	4.6	4.1	^3^0.093
	Gallbladder diameter, mean (cm)	3.3	3.3	^3^0.870
	Pericholecystic fluid, n(%)	17(31.5)	18(28.6)	^1^0.732
Intraoperative findings	Edema in GB wall, n(%)	32(59.3)	38(60.3)	^1^0.907
	Hydropic GB, n(%)	38(70.4)	38(60.3)	^1^0.256
	Adhesions, n(%)	28(51.9)	30(47.6)	^1^0.648
	Gangrenous GB, n(%)	11(20.4)	8(12.7)	^1^0.262
	Perforated GB, n(%)	7(13)	4(6.3)	^1^0.222
	Subtotal cholecystectomy, n(%)	6(11.1)	1(1.6)	^4^0.030^*^
	Parkland score, mean	3.3	2.8	^3^0.008^*^

The conversion rates to open cholecystectomy, complication rates, intensive care treatment rates, readmission rates, and mortality rates did not show statistically significant differences between the surgeons both before and after three days (p>0.05). Among all patients, there were two reported complications. One case involved a perihepatic collection after surgery, which was successfully treated with percutaneous drainage and endoscopic retrograde cholangiopancreatography. The other case presented with obstructive jaundice managed with endoscopic retrograde cholangiopancreatography. The mean operative times were similar between the surgeons both before and after three days. However, after three days, the operative time was longer than the first three days in the HVS group (p=0.011), while the other parameters related to operative time showed no significant differences (p>0.05). The postoperative hospital stay was 3.1 days in the LVS group and 1.9 days in the HVS group (p=0.087). Specifically, for the first three days, the postoperative hospital stay was 1.8 days in the LVS and two days in the HVS group (p=0.811). Beyond three days, the postoperative hospital stay was 3.7 days in the LVS and 1.9 days in the HVS group, which showed a statistically significant difference (p=0.040) (Table 4).

**Table 4:** Intraoperative and postoperative outcomes among the surgeons ¹ Fisher’s exact test, ^2 ^Mann Whitney U test, ^3 ^Pearson chi-square test, * p<0.05 significance

		Low-volume surgeons (n:54)	High-volume surgeons (n:63)	p-value
Conversion to open cholecystectomy, n (%)	Total	54/2(3.7)	63/2(3.2)	^1^1.000
	≤3 days	18/0	31/2(6.5)	^1^0.526
	>3 days	36/2(5.6)	32/0	^1^0.494
	p	^1^0.547	^1^0.238	
Complications, n (%)	Total	54/2(5.1)	63/0	^1^0.211
	≤3 days	18/0	31/0	
	>3 days	36/2(5.6)	32/0	^1^0.494
	p	^1^0.547		
Operative time, mean (minute)	Total	84.6	73.4	^2^0.059
	≤3 days	77.7	65.1	^2^0.355
	>3 days	88.1	81.5	^2^0.279
	p	^2^0.130	^2^0.011^*^	
Intensive care treatment, n(%)	Total	54/3(5.6)	63/0	^1^0.095
	≤3 days	18/0	31/0	
	>3 days	36/3(8.3)	32/0	^1^0.241
	p	^1^0.543		
Readmission, n(%)	Total	54/3(5.6)	63/2(3.2)	^1^0.661
	≤3 days	18/0	31/2(6.5)	^1^0.526
	>3 days	36/3(8.3)	32/0	^1^0.241
	p	^1^0.543	^1^0.238	
Mortality, n(%)	Total	54/1(1.9)	63/0	^1^0.462
	≤3 days	18/0	31/0	
	>3 days	36/1(2.8)	32/0	^1^1.000
	p	^1^1.000		
Total hospital stay, mean (days)	Total	4.6	3.2	^2^0.130
	≤3 days	2.4	2.5	^2^0.901
	>3 days	5.6	3.9	^2^0.155
	p	^2^0.001^*^	^2^0.001^*^	
Postoperative hospital stay, mean (days)	Total	3.1	1.9	^2^0.087
	≤3 days	1.8	2	^2^0.811
	>3 days	3.7	1.9	^2^0.040^*^
	p	^2^0.080	^2^0.782	
Pathology (Acute cholecystitis), n(%)		18(33.3)	19(30.2)	^3^0.713

## Discussion

In our study, we found similar results among the groups based on symptom duration. The rate of conversion to open cholecystectomy, complication rates, intensive care treatment rates, readmission rates, and mortality rates showed no statistical differences. The only notable difference observed was a longer operative time for patients with symptom duration exceeding three days. These findings demonstrate that symptom duration should not be taken into consideration when deciding on the ELC. In recent years, performing ELC without adhering to the 72-hour rule in AC has been supported by the guidelines. For instance, the Tokyo 2018 guideline recommends ELC within seven days, regardless of symptom duration, as long as the patient can tolerate the surgery [10]. Similarly, the World Emergency Surgery Association guideline suggests ELC within 10 days from the onset of symptoms [11]. 

In the literature, several studies support performing ELC without considering symptom duration [7,12,17-20]. However, studies still support the 72-hour rule or limiting the symptom duration to seven days [6,21,22]. Most of these studies lack subgroup analysis, with only two out of the mentioned nine studies performing such analysis. Dimartino et al. [12] conducted a study where higher conversion rates to open cholecystectomy and intraoperative complications were observed after seven days from symptom onset. However, when hospitals were categorized into high- and low-volume centers based on their early laparoscopic case volumes in one year, the adverse outcomes after seven days were seen primarily in low-volume centers, while no significant difference was observed in high-volume centers. As a result, ELC was recommended regardless of symptom duration in high-volume centers, but not after seven days in low-volume centers. Another study by Chia et al. [17] performed a subgroup analysis based on age and recommended ELC specifically for the elderly population (aged 75 and above). In the remaining seven studies, subgroup analysis was not conducted, although the materials and methods sections indicated that the surgeons had experience in laparoscopy. Only one study mentioned that the surgeons had high volumes of laparoscopic surgeries [18]. Wiggins et al. [22] performed subgroup analysis in their study using data collected from various hospitals in the United Kingdom, recommending ELC within the first three days. The hospitals were divided into three groups based on the number of emergency cholecystectomy cases they performed. However, this study focused on the symptom range for each hospital based on volume and did not evaluate postoperative outcomes based on hospital volume. Additionally, information regarding the laparoscopic experience or volume of the surgeons was not provided. Furthermore, there are studies in the literature that specifically examine the impact of surgeon experience in ELC for AC. These studies generally report lower rates of conversion to open cholecystectomy in experienced laparoscopic surgeons, while complication rates remain similar among the surgeons [13-15].

In our study, we conducted a subgroup analysis based on the surgeon volume. All surgeons working in different general surgery departments in our clinic have received comprehensive training in laparoscopic surgery. Surgeons with other specialist interests have significant experience performing various laparoscopic procedures such as hernia repair, bariatric surgery, gastric and colon surgery, and endocrine surgery throughout the year. Therefore, we considered the number of cholecystectomy surgeries they performed annually to categorize the surgeons. The preoperative characteristics and intraoperative findings among the surgeons were similar. However, there were higher numbers of gangrenous and perforated gallbladders in the LVS compared to the HVS. Additionally, the rates of subtotal cholecystectomy and Parkland scores were significantly higher in the LVS. When analyzing the results, there were no significant differences in intraoperative and postoperative outcomes among the surgeons, except for a longer hospital stay observed in the LVS. We attribute this difference to the higher gangrenous and perforated cholecystitis cases, as well as the increased incidence of subtotal cholecystectomy and higher Parkland scores seen in the LVS.

Study limitations

Our study has certain limitations that need to be acknowledged. Firstly, it is important to note that this study is retrospective, which inherently poses challenges in avoiding patient selection bias. Secondly, the sample size of our study is relatively small, which may limit the generalizability of the findings. Therefore, to confirm and strengthen our findings, it is recommended that future studies incorporate larger sample sizes and utilize prospective study designs. These measures will help provide more robust evidence and enhance the validity of our conclusions regarding the benefits of ELC in cases of AC.

## Conclusions

This study examined symptom duration, surgeon volume, and the detailed relationship between these factors in early laparoscopic cholecystectomy for acute cholecystitis. It demonstrated that symptom duration longer than 72 hours should not be a determining factor. In recent years, the trend of not complying with the 72-hour rule on symptom duration is also supported by guidelines. Moreover, the study affirms that surgeon volume does not significantly influence outcomes for surgeons skilled in laparoscopic procedures. Additionally, no substantial correlation was observed between surgeon volume and symptom duration. Based on our study findings, ELC may be recommended for AC, irrespective of symptom duration or surgeon volume, for surgeons with advanced laparoscopic surgical training. It is recommended that future research confirm and consolidate these results by using prospective studies with larger sample sizes.
